# 

**Published:** 1879-11

**Authors:** 


**Notice.**—The Books and Surgical Instruments belonging to the Free Medical and Surgical Dispensing Association of Buffalo will be sold at *Public Auction*, on Saturday, November 8th, 1879, at the Museum of the Buffalo Medical College.

M. B. FOLWELL, M. D.,

President Board of Directors.

